# Dose-dependent behavioural fever responses in desert locusts challenged with the entomopathogenic fungus *Metarhizium acridum*

**DOI:** 10.1038/s41598-018-32524-w

**Published:** 2018-09-21

**Authors:** Lisa M. Clancy, Rory Jones, Amy L. Cooper, Gareth W. Griffith, Roger D. Santer

**Affiliations:** Institute of Biological, Environmental, and Rural Sciences, Aberystwyth University, Penglais Campus, Aberystwyth, Ceredigion SY23 3FG United Kingdom

## Abstract

Behavioural fever is a common response to immune challenge in ectotherms and confers survival benefits. However, costs accrue rapidly as body temperature rises. Thus, the magnitude of adaptive fever responses might reflect the balance of costs and benefits. We investigated behavioural fever in desert locusts, *Schistocerca gregaria*, infected with the entomopathogenic fungus *Metarhizium acridum*. We first tracked the time course of behavioural fever in infected locusts, demonstrating that body temperatures rose on the day following inoculation (day 1), and reached peak intensity on the day after that (day 2). Subsequently, the magnitude of fever responses varied during a day, and locusts tended to exhibit high-intensity fever responses in the mornings when basking was first possible. We speculate that this may have resulted from increased fungal load caused by unimpeded growth overnight when locusts could not fever. We next inoculated locusts with different *M*. *acridum* doses ranging from 0 to ca. 75,000 conidia. The magnitude of their behavioural fever responses on day 2 post-inoculation was positively related to fungal dose. Thus, we demonstrate dose-dependency in the behavioural fever responses of desert locusts and suggest that this may reflect the adaptive deployment of behavioural fever to minimize costs relative to benefits.

## Introduction

Fever body temperatures are an adaptive response to infection in many species, and they can be achieved by physiological and/or behavioural means such as basking^1,2^. Ectotherms rely exclusively on behavioural strategies to achieve fever, and these have been described across a diverse range of taxa including reptiles, amphibians, and insects^2^. Behavioural fever can decrease mortality and morbidity in infected animals^3–6^. This occurs because high body temperatures are suboptimal for pathogen growth^5,7^ and increase the mortality of some pathogens^6^; high body temperatures also enhance several aspects of host immune function^8,9^.

Despite their benefit for fighting infection, fever body temperatures are also costly^10^. Elevated body temperatures have been associated with increased metabolic rate^11,12^, reduced growth rates^13^, as well as defects in egg development^14^. Furthermore, basking to achieve fever body temperatures confers additional costs in terms of missed feeding and mating opportunities, and increased predation risk^15^. These costs accrue quickly as body temperature rises, such that the temperature-fitness curves of healthy animals are often asymmetrical, with fitness declining more steeply with increasing temperature than with decreasing temperature^16^. As a consequence, and because of their inability to thermoregulate perfectly, healthy ectotherms tend to adopt body temperatures slightly below the theoretical optimum, in order to avoid the disproportionate costs of unintentionally straying into higher body temperatures^16^. Sick individuals likely face a similar requirement to balance costs and benefits and avoid excessively high fever body temperatures, especially since behavioural fever responses are often unable to fully clear infection^5,17^ (but see^6^). In these cases, fever body temperatures might be expected to be modulated according to the severity or type of infection^18^.

In support of the adaptive deployment of fever responses, the occurrence and magnitude of behavioural fever are known to vary within and between insect species^19^. Crickets, *Acheta domesticus*, elicited behavioural fever responses when infected with thermo-susceptible parasites (e.g. *Rickettsiella grylli*), but not thermotolerant ones (e.g. *Serratia marcescens*, and the parasitoid fly *Ormia ochracea*)^20^. House flies, *Musca domestica*, infected with a higher dose of the fungus *Beauveria bassiana* exhibited higher-intensity fever responses than flies infected with a lower dose, putatively limiting fever costs^21^. The physiological fevers of humans may also vary in magnitude according to the severity of infection^2^. Thus, the adaptive deployment of fever may be a widespread phenomenon.

In this study, we investigate whether desert locust, *Schistocerca gregaria*, behavioural fever responses differ in intensity according to the severity of *Metarhizium acridum* infection. *Metarhizium acridum* is a specialist pathogen of locusts and grasshoppers and the basis of commercial biopesticides used for their control^22^. Whilst numerous laboratory and field trials have demonstrated the efficacy of these biopesticides, the speed of kill following application is highly variable (see^23^, and references therein). The prevailing theory is that environmental temperature leads to this variability, by allowing or preventing effective behavioural fever responses^5^. Thus, additional knowledge of locust behavioural fever responses to *Metarhizium* infection may also help inform the application of biopesticides for locust control.

## Results

### The behavioural fever response to infection with *Metarhizium acridum*

**Figure 1:**
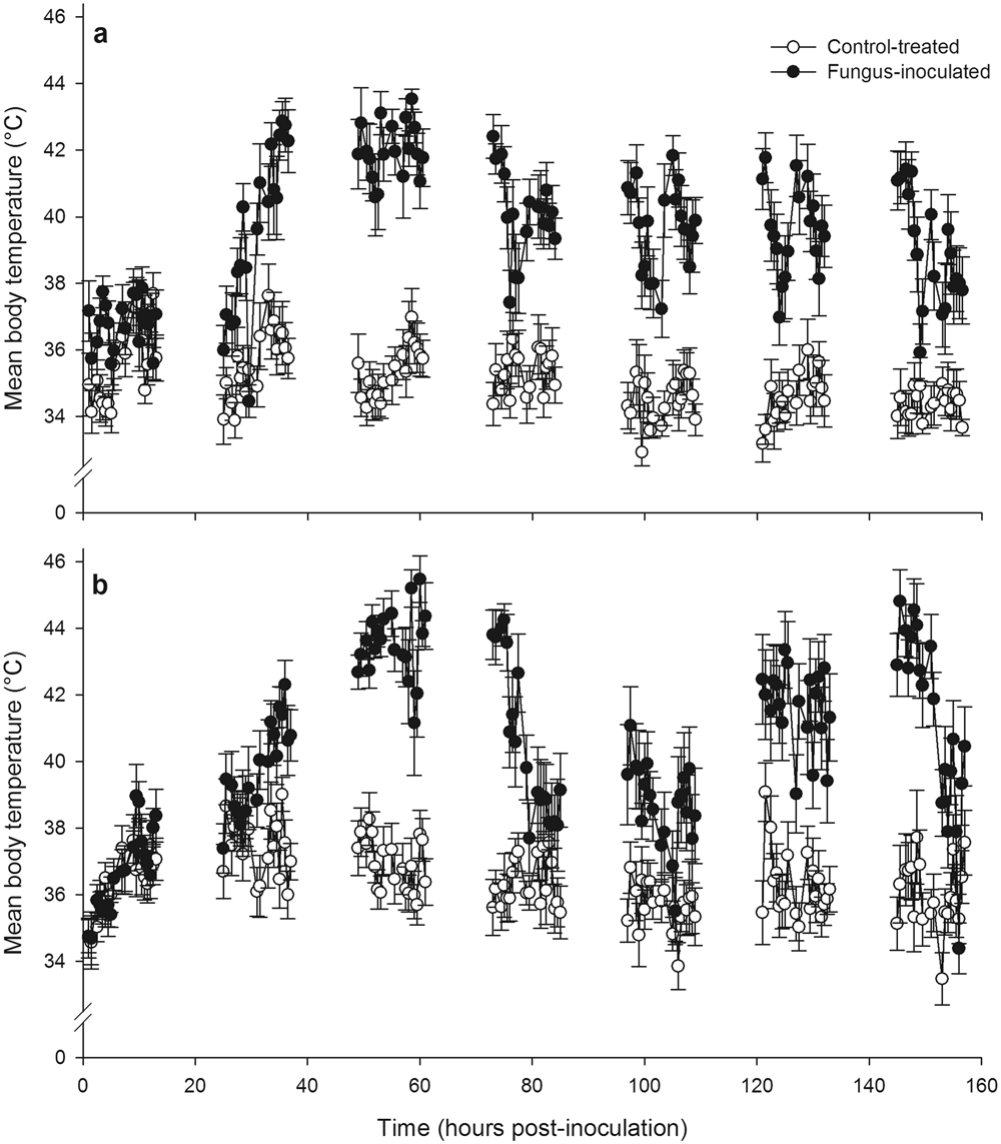
The time course of behavioural fever in desert locusts inoculated with *Metarhizium acridum*. Plots (**a**,**b)** show the results of two repeats of the same experiment. In each experiment, the body temperatures of 12 fungus-inoculated (filled circles) and 12 control-treated (open circles) locusts was recorded by infrared thermography at 30-minute intervals during the operation of a thermal gradient. Data points show the mean ± SEM body temperature across the 12 locusts in each treatment group at each sampling point. Clusters of sampling points, therefore, represent subjective days 0 to 6 of the experiment, and intervals between them the subjective nights in which an ambient temperature of 28 °C was maintained. The fever responses of fungus-inoculated locusts were most intense on day 2, 49–61 hours post-inoculation (filled circles). On subsequent days the first sampling points of the day often yielded high mean body temperatures which tended to decline at subsequent measurements.

We first examined the time course of the behavioural fever response in *M*. *acridum*-infected and control-treated desert locusts housed in aluminium cages equipped with overhead basking lamps. Basking lamps operated during the 12 h photophase, during which locusts thermoregulated freely; during the 12 h scotophase an ambient temperature of 28 °C was maintained. We monitored each locust’s temperature using infrared thermography at regular intervals during the photophase for seven days following inoculation. We carried out two such experiments, each using a cohort of 12 locusts per treatment group that were housed in groups of six (see Fig. 1).

We found a significant effect on locust body temperature of fungal treatment, and a significant interaction between fungal treatment and day post inoculation (GEE: Treatment: Wald Χ^2^_1_ = 345.701, p < 0.001; Day: Wald Χ^2^_6_ = 1048.149, p < 0.001; Treatment*Day: Wald Χ^2^_6_ = 993.322, p < 0.001). Although there was a significant difference between our two experiments, and a significant interaction between day and experiment, there was no significant interaction between experiment and treatment, or between experiment, treatment, and day (Experiment: Wald Χ^2^_1_ = 22.837, p < 0.001; Experiment*Day: Wald Χ^2^_6_ = 65.838, p < 0.001; Experiment*Treatment: Wald Χ^2^_1_ = 2.062, p = 0.151; Experiment*Treatment*Day: Wald Χ^2^_6_ = 11.924, p = 0.064). Thus, despite differences between our experiments, fungal infection had a significant effect on locust body temperature that varied over the seven-day period post-inoculation in a similar way in both experiments.

Over the course of our experiments, the mean body temperature of control locusts at each sampling point fluctuated in the range ca. 33–39 °C (see Fig. 1). Meanwhile, climbing body temperatures of *M*. *acridum*-infected locusts were strongly evident during day 1 post-inoculation (26–37 hours post-inoculation) and their body temperatures peaked on day 2 post-inoculation (49–61 hours post-inoculation). On this day infected locusts adopted mean body temperatures in the range ca. 40.5–45.5 °C. To statistically compare body temperatures on day 2 post-inoculation, because body temperatures fluctuated relatively little during that day (see Fig. 1), we calculated a mean body temperature across all temperature sampling points on day 2 for each individual locust. We found significant differences in this measurement between control and fungus-infected locusts, and between experiments, but no significant interaction between fungal treatment and experiment (GLM: Treatment: F_1,44_ = 540.886, p < 0.001; Experiment: F_1,44_ = 30.247, p < 0.001; Treatment*Experiment: F_1,44_ = 0.014, p = 0.907; Fig. 1). Over the remaining days of the experiment, infected locusts tended to adopt higher body temperatures than controls, though these fluctuated over the course of a day (see Fig. 1). It was notable during this period that locusts often selected higher temperatures at the start of each day, and we hypothesized that this may have been a response to fungal growth overnight (see Fig. 1).

### Dependence of behavioural fever body temperatures on *Metarhizium acridum* dose

We next inoculated locusts with varying doses of *M*. *acridum* equating to 0, and ca. 750, 7,500, 15,000 and 75,000 conidia (asexual spores), in order to examine the dose-dependence of the behavioural fever response. Locusts were housed as in our fever time course experiments, but in these experiments basking lamps were in operation for 10 h within the 12 h photophase. We assayed individual locusts’ fever body temperatures via infrared thermography at regular intervals during basking lamp operation on day 2 post-inoculation, at the height of the fever response identified in our first experiment (see Fig. 1). We conducted two such experiments, each on a cohort of 12 locusts per treatment.

**Figure 2:**
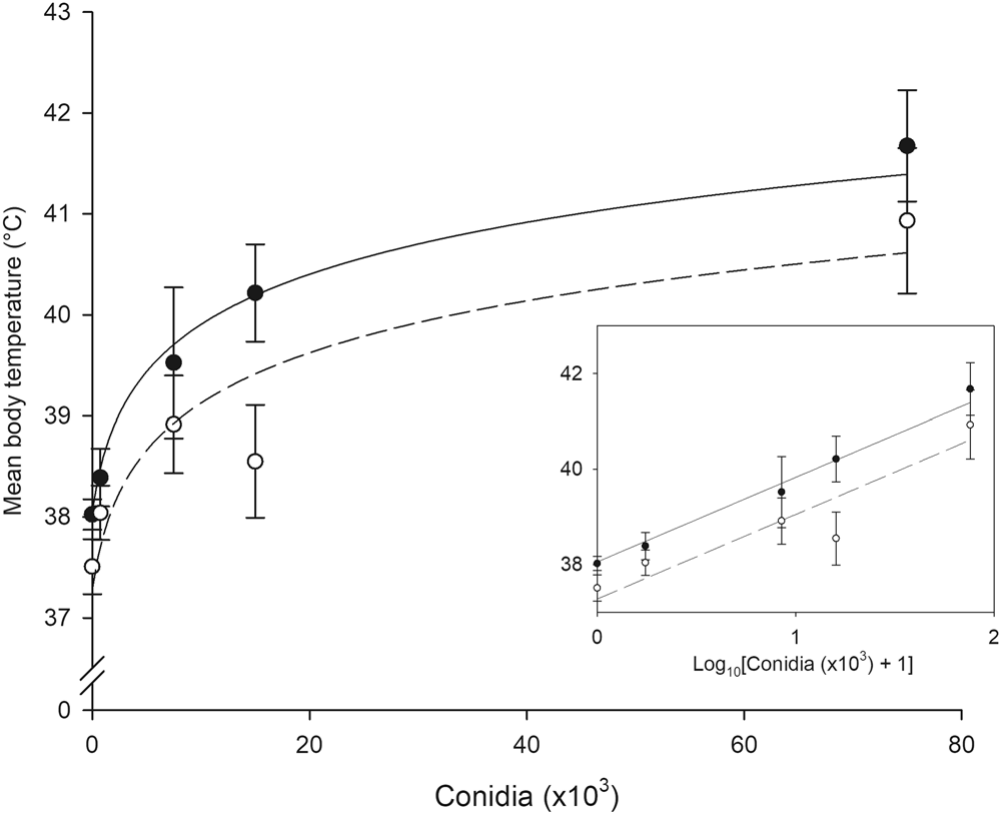
The relationship between fungal dose and the intensity of behavioural fever. Filled and open circles indicate the results of two repeats of the same experiment. In each experiment, cohorts of 12 locusts were inoculated with one of five fungal doses ranging from 0 to ca. 75,000 fungal conidia. For each locust, body temperatures were measured at 14 temperature sampling points on day 2 post-inoculation, and these measurements were averaged to indicate the intensity of fever on that day. Data points show the mean ± SEM body temperature across the 12 locusts in each cohort. Locust body temperatures were significantly linearly related to log_10_-transformed fungal doses (inset; see results). These significant relationships are plotted for each experiment (solid and dashed lines), against both log_10_-transformed (inset) and untransformed fungal doses (main figure).

Mean body temperatures calculated across all measurements of an individual on day 2 scaled linearly with log_10_-transformed fungal dose (see Fig. 2, inset), reflecting an initially steep, but slowing, increase in body temperature as fungal dose increased (see Fig. 2). Statistical analysis of these trends identified a significant difference between experiments, and a significant dependence of body temperature on log_10_-transformed fungal dose in the complete dataset (GZLM: Experiment: Wald Χ^2^_1_ = 6.548; p = 0.011; Dose: Wald Χ^2^_1_ = 62.361; p < 0.001; Fig. 2). In both experiments, locusts inoculated with cottonseed oil only adopted body temperatures consistent with those of control locusts in Fig. 1; likewise, those inoculated with the greatest dose of *M*. *acridum* adopted fever body temperatures consistent with those in Fig. 1. The magnitude of the fever response was intermediate with intermediate fungal doses. Thus, there was a clear and significant dependence of behavioural fever intensity on fungal dose.

## Discussion

After locusts were inoculated with *Metarhizium* fungus (on day 0), their behavioural fever response began on day 1 post-inoculation and peaked on day 2 post-inoculation, consistent with previous reports^24^. The intensity of fever varied over the course of subsequent days, and was often greatest in the morning when basking was first possible. Peak behavioural fever temperatures on day 2 post inoculation were significantly positively related to fungal dose, with more severe infections inducing more intense fever responses. These lines of evidence demonstrate that behavioural fever responses are modulated according to fungal dose in locusts infected with *Metarhizium* fungus.

In order to infect an inoculated locust, *Metarhizium* conidia must germinate and penetrate the cuticle, leading to a systemic infection of haemocoel-specific hyphal bodies^25^. When desert locusts were topically inoculated and kept at 28 °C, hyphal bodies were present in the haemolymph after ca. 48 hrs, since transfer of haemolymph from inoculated to uninfected desert locusts at two or three days post-inoculation caused infection, but was not effective one day post-inoculation^26^. The conidia of *Metarhizium acridum* isolates ARSEF 324 and 7486 are relatively tolerant of exposure to high temperatures^27,28^, but germination of ARSEF 324 was impaired at constant temperatures ≥ 38 °C^29^ and delayed by brief exposure to temperatures of 47 °C^27^. In our experiments, control and fungus-inoculated locusts adopted body temperatures in the range 34–38 °C following inoculation, before experiencing nighttime temperatures of 28 °C ca. 12 h later. Despite any delay in germination due to this thermal regime, the ramp-like increase in locust body temperature from about 24 hours post-inoculation observed in our and previous experiments^24^ would approximately coincide with the onset of cuticle penetration and subsequent exponential growth of fungus in the haemolymph. This provides an indirect line of evidence that the intensity of behavioural fever responses are proportional to the extent of infection. The availability of immune-eliciting cues that could elicit behavioural fever during cuticle penetration is supported by the observation that total haemocyte counts were already elevated in desert locusts at one day post-inoculation^26^. Injections of immune-eliciting compounds directly into circulation cause intense fever responses evident at 24 hours post-treatment^30^, so detailed time courses of such fevers may help reveal the extent to which fever development is determined by the build-up of immune elicitors.

A second indirect line of evidence suggesting that the intensity of behavioural fever responses is proportional to the extent of infection comes from locust body temperatures later during fever time courses. *In vitro*, *Metarhizium acridum* ARSEF 324 hyphal growth ceases at temperatures ≥38 °C, but resumes when cultures are transferred to 28 °C^29^. Similarly, the behavioural fever responses of infected locusts impede *Metarhizium* growth but do not clear the infection^5,17^. In the course of our experiments, basking lamps were turned off overnight and the ambient temperature was 28 °C, during which *Metarhizium* growth would again have been possible and fungal load may have increased. As in our experiments, intense fever responses once basking was possible have been observed in house flies, *Musca domestica*, infected with the fungal entomopathogen, *Beauveria bassiana*, and this has been explained as a response to increased fungal load during a period in which behavioural fever was not possible^21^.

Our most compelling and direct evidence for the plasticity of fever responses comes from our demonstration that locusts challenged with higher doses of *Metarhizium* fungus exhibited higher-intensity fever responses than locusts infected with lower doses. This was robustly demonstrated in two separate experiments. A similar dose-dependency of behavioural fever has also been observed in house flies challenged with *B*. *bassiana*^21^, and a relationship between physiological fever intensity and the severity of infection has been suggested in humans^2^. Thus, the property of dose-dependency may be a feature of fever responses generally, and behavioural fever responses particularly, across taxonomic groups of hosts and pathogens. Interestingly, we found that fever intensity related linearly to log_10_-transformed fungal doses applied topically; if the same relationship occurred with circulating fungal loads, this would predict a ramp-like increase in fever intensity in response to exponential fungal growth.

A proximate explanation for the dose-dependency of behavioural fever responses has been proposed by Anderson *et al*. (2013)^21^ who suggested that the intensity of fever responses is related to the amount of immune elicitors presented by the invading pathogen. Such an explanation is consistent with experimental data for locusts which demonstrate that behavioural fever responses are triggered by PAMPs (pathogen associated molecular patterns), e.g. the microbial cell wall components laminarin and lipopolysaccharide^30^, the quantity of which in circulation would be expected to correlate with fungal load. However, *Metarhizium anisopliae* hyphal bodies are able to hide their PAMPs from the insect immune system via production of a collagenous coat by the rapid expression of the *Mcl1* gene on contact with haemolymph^31^. Since total haemocyte counts were already elevated in desert locusts at one day post-inoculation before hyphal bodies were present in haemolymph^26^, immune-eliciting cues likely also arise during cuticle penetration (e.g. from fungal enzymes or degraded cuticle). It is conceivable then that immune-eliciting cues are most available early in infection, leading to the observed peak fever intensities on the second day post-inoculation, though it is by no means certain what these cues are. Interestingly, total haemocyte counts have been observed to peak and decline on a similar timescale to fever responses observed in this study^26^.

Given the cost of maintaining fever responses, ultimate explanations for the dose-dependency of fever might also be hypothesised. Antipyretics are released during fever in both ectotherms and endotherms, which act to prevent body temperatures from rising to dangerous levels, indicating a highly conserved function of keeping the costs of fever in check^2^. Modulation of fever responses according to pathogen dose may allow insects to minimise the costs of fevering by exhibiting the lowest-intensity response required to control infection^21^. *In vitro* studies of *Metarhizium* show that temperature increases above the optimum gradually restrict growth before preventing it^5,32^, and studies of infected locusts show that restricting locust body temperatures and interrupting their fever bouts impedes their ability to contain *Metarhizium* infection^5,17,33^. Thus, it seems likely that less intense behavioural fever responses would be less effective at restricting *Metarhizium* growth. However, since locust behavioural fever responses control *Metarhizium* growth but appear unable to clear infection^5,17^, less effective fever responses may be adequate for delaying the development of a light infection that is inflicting limited costs on the host. This is because more intense fevers would incur greater costs, but could not achieve greatly increased benefits due to their inability to actually clear the infection^5,17^.

Our results have implications for the future study of behavioural fever responses in insects and other animals. Firstly, like Hunt *et al*. (2011)^24^, our experiments employed infrared thermography and a relatively high frequency of temperature sampling points in time. This approach contrasts with earlier studies that gauged the subject animal’s temperature by recording its position on a calibrated thermal gradient or by measuring the ambient temperature in its immediate vicinity, and/or that used single, ‘snap-shot’ measurements rather than time courses^7,13,20,30^. The greater sensitivity offered by methods like ours enabled Hunt *et al*. (2011)^24^ to detect fever responses earlier post-inoculation than had been possible, a finding which we now support. Our observation that fever responses are also variable in their magnitude adds further weight to the argument that a consistent, sensitive methodology is essential in order that studies can be compared and variations in fever responses between species of host and pathogen, and across pathogen doses, can be assessed. Secondly, we followed the practice of some previous studies in using male locusts to control fungal inoculant dose in relation to body size (c.f.^24,30,34^). However, fungal infection affects traits related to reproduction in both male and female locusts^4,35,36^; and fever temperatures themselves have been shown to affect male mating behaviours (but not survival or female fecundity)^37^, and to cause shifts towards the solitarious behavioural phase state in offspring^38^. Therefore, it is important that future studies focus on males, females, and offspring for a complete understanding of the costs and benefits that may drive the adaptive deployment of fever responses (see also^37^).

The behavioural fever response (in conjunction with ambient temperature) is one of the key constraints in the use of fungal entomopathogens for the biocontrol of insect pest species^22,39^. As such, efforts are being made to identify^32,33^ or bioengineer^40^ fungal strains that are better able to tolerate fever temperatures, or that can inhibit the fever response outright^34^. At the same time, models have been created to predict the efficacy of fungal biopesticide applications under varying ambient temperature conditions^41^. A better understanding of the exact fever temperatures adopted by infected locusts, according to the efficiency with which they are dosed and the biopesticide they are dosed with, may help to fine-tune these models and increase their power to inform control operations. Such technologically enhanced pest management may be of particular importance given the potential for rising global temperatures to influence the interaction between insect pests and their pathogens^42,43^.

## Methods

### Culture and maintenance of fungi and locusts

*Metarhizium acridum* was obtained from the Agricultural Research Service Collection of Entomopathogenic Fungi (ARSEF) Culture, Cornell University, Ithaca, NY. We used *M*. *acridum* isolate ARSEF 324 (originating from Australia), in our fever time course experiments, and *M*. *acridum* isolate ARSEF 7486 (originating from Niger), in our dose-response experiments^28^. Both are virulent isolates of *M*. *acridum*, and are the active ingredients in Green Guard ® and Green Muscle ® biopesticides, respectively^28,44,45^. Fungal cultures were grown on Potato Dextrose Agar (Lab M Ltd., Lancashire, UK), maintained in incubators at 28 **°**C. The incubator used for most fungal cultures provided continuous light, but that used to grow ARSEF 7486 for our second dose-response experiment did not provide light.

Adult desert locusts, *S*. *gregaria*, were purchased from Blades Biological Ltd. (Edenbridge, Kent, UK). Our intention was to use male locusts only, to control for fungal inoculant dose in relation to body size/mass, as female locusts are larger than male locusts (c.f.^24,30,34,35^). However, a small number of females were included in our first dose-response experiment. Locusts were housed in 33 × 20 × 41 cm aluminium cages with a plexiglass front and perforated mesh floor. Cages were kept in a controlled-temperature room at 28 °C, with a 12 h light: 12 h dark photocycle. Locusts were provided with water and wheat bran *ad libitum*, and fresh wheat seedlings three times per week. These were provided on the cage floor.

### Fungal inoculations and control treatments

To prepare fungal inoculants, 10 ml of cottonseed oil (Sigma Aldrich, Dorset, UK) was added to the surface of 7-day old fungal cultures. Conidia were dislodged using a sterile spreader and the suspension was transferred to a sterile 25 ml bottle. The suspension was then filtered through four layers of sterile muslin to filter out mycelia and clumps of conidia, centrifuged at 3000 rpm for 3 min, re-suspended in fresh cottonseed oil, and then placed in a sonicating water bath (15 °C for 5 min). Conidial concentration was determined using a Neubauer haemocytometer and adjusted to 3.75 × 10^7^ per ml.

In order to examine the time course of behavioural fever, locusts were inoculated with 2 μl of conidial suspension (ca. 75,000 spores) by topical application under the pronotal shield using a micro-syringe (VICI ®, Baton Rouge, Louisiana, USA) (c.f.^34,35,46^). Controls were treated with 2 μl of cottonseed oil alone.

In order to examine the dose dependence of the behavioural fever response, locusts were inoculated with 2 μl of the full strength fungal suspension (ca. 75,000 conidia), or 1:5 (ca. 15,000 conidia), 1:10 (ca. 7,500 conidia), or 1:100 (ca. 750 conidia) dilutions of it. Controls were again treated with 2 μl of cottonseed oil alone. Inoculants were applied topically under the pronotal shield using a 0.5–10.0 μl micro-pipette (Accumax Pro, Accumax Lab Technology, Gandhinagar, India).

### Assaying behavioural fever

Locusts were housed in cages (as described above), with a 5 mm wire mesh for climbing attached to the back wall of the cage. Each cage was equipped with a 60 W incandescent reflector light bulb placed on top of the cage above a 5 mm wire mesh lid, in order to provide a thermal gradient for basking. These bulbs operated for either 12 h (fever time course experiments) or 10 h (dose-response experiments) during the photophase of the 12 h: 12 h photocycle in the experimental room, and provided a thermal gradient of ca. 30–50 °C in each locust cage. When basking lamps were not in operation, an ambient temperature of 28 °C was maintained in locust cages.

We conducted two experiments investigating the time course of behavioural fever. In each experiment, 12 male locusts were inoculated with *Metarhizium* and 12 male locusts treated as controls. Following inoculation, locusts of each treatment group were split across two cages (thus, six locusts of the same treatment group per cage). Our naming convention is that this first day of the experiment is day 0. A FLIR i50 thermal imaging camera (FLIR Systems, Inc., Wilsonville, Oregon, USA) was used to measure locust body temperatures, following Hunt *et al*. (2011)^24^. Emissivity was assumed to be 0.97 as in other insects^47^. Infrared data were recorded at 30-min intervals between 09.00 and 14.00 and 15.30 and 19.30 from days 0 to 6 (1–157 h post-inoculation). On each day light/basking heat-on was at 08.00, and light/basking heat-off at 20.00. When provided, wheat seedlings were added to the cage between 14.00 and 15.30 since locusts tended to immediately descend to the cage floor and feed, which would otherwise have affected body temperature measurements; wheat bran and water were available *ad libitum*. The infrared camera focal point was directed at the thorax area of each locust (c.f.^24^). Wherever possible, we focused on the dorsal thorax, though locusts exhibiting fever sometimes hang upside down under the heat bulb and in these cases the camera focal point was directed at the ventral thorax.

The dose-dependence of behavioural fever was also investigated in two experiments. In each experiment, a total of 12 locusts were inoculated per fungal dose and housed within the same cage for the duration of each experiment (thus, 12 locusts per cage). Infrared temperature data were then recorded at 30-min intervals from 09.00 to 12.00 and from 14.00 to 17.00. In these experiments light-on/off in the experimental room was 07.00 and 19.00, respectively, and basking lamp heat-on/off for each locust cage was 08.00 and 18.00, respectively. Wheat seedlings were added to the cage between 12.00 and 14.00; wheat bran and water were available *ad libitum*. In our first experiment of this kind the total sample was 51 males and 9 females, distributed across treatments; in our second experiment the total sample size was 60 males.

### Statistical analyses

All statistical analyses were conducted using SPSS version 23 (IBM Corp., Armonk, NY, USA).

In order to analyse the time course of locust body temperature measurements, a repeated-measures approach was adopted using Generalised Estimating Equations (GEE) assuming a normal distribution and identity link function. The relatedness of body temperature measurements from the same individual locust at the 140 temperature sampling time points post-inoculation was modelled using the first-order autoregressive [AR(1)] covariance structure. The AR(1) covariance structure is considered suitable for longitudinal data sets as it accounts for a reduction in the correlation between measurement times as time points get further apart. ‘Treatment’, ‘day’ and ‘experiment’ were included as factors, and all interaction terms were included. Subsequently, in order to analyse body temperatures on day 2 post-inoculation when fever was most intense and body temperatures relatively constant, we computed a mean body temperature for each locust across all temperature sampling points on that day and analysed that measurement as the dependent variable using a General Linear Model (GLM). In this analysis, ‘treatment’ and ‘experiment’ were factors, and an interaction term was included.

In order to analyse the dose-dependence of behavioural fever, we focused on locust temperatures recorded during the 14 measurements on day 2 post-inoculation (the peak of the fever response determined from our fever time course experiments). For each locust we computed the mean body temperature across all 14 measurements on that day and analysed these as the dependent variable. Fungal doses expressed as approximate numbers of conidia (x10^3^) were transformed by log_10_(x + 1) to linearise their relationship with locust body temperature. We then implemented a Generalized Linear Model (GZLM) assuming a normal distribution and identity link function. Log_10_-transformed ‘fungal dose’ was a covariate, ‘experiment’ a factor, and their interaction was included. Because the interaction term was not significant, it was removed and the analysis repeated (the ‘experiment’ effect was not significant in the full factorial model, but became so after removal of the interaction term). This main effects model is reported in Results. Because a small number of females were included in the experiment 1 sample, we took steps to ensure that their inclusion did not influence the trends we report. The effect of ‘fungal dose’ was significant for both the experiment 1 dataset and the combined dataset, with and without the data for the nine females. The effect of ‘experiment’ was no longer significant for the combined dataset when those nine females were omitted.

## Electronic supplementary material

Dataset 1

## Data Availability

All data generated and analysed during this study are provided in Supplementary Dataset 1.
